# The Hyperledger fabric as a Blockchain framework preserves the security of electronic health records

**DOI:** 10.3389/fpubh.2023.1272787

**Published:** 2023-11-28

**Authors:** Muhammad Hasnain, Fahad R. Albogamy, Saeed S. Alamri, Imran Ghani, Bilal Mehboob

**Affiliations:** ^1^Department of Computer Science, Lahore Leads University, Lahore, Pakistan; ^2^Turabah University College, Computer Sciences Program, Taif University, Taif, Saudi Arabia; ^3^Electrical Engineer Consultant, Jeddah, Saudi Arabia; ^4^Department of Computer and Information Sciences, Virginia Military Institute, Lexington, KY, United States; ^5^Department of Software Engineering, Superior University, Lahore, Pakistan

**Keywords:** anonymity, Ethereum, blockchain, throughput, latency, health records

## Abstract

The Hyperledger Fabric (HF) framework is widely studied for securing electronic health records (EHRs) in the healthcare sector. Despite the various cross-domain blockchain technology (BCT) applications, little is known about the role of the HF framework in healthcare. The purpose of the systematic literature review (SLR) is to review the existing literature on the HF framework and its applications in healthcare. This SLR includes literature published between January 2015 and March 2023 in the ACM digital library, IEEE Xplore, SCOPUS, Springer, PubMed, and Google Scholar databases. Following the inclusion and exclusion criteria, a total of 57 articles emerged as eligible for this SLR. The HF framework was found to be useful in securing health records coming from the Internet of Medical Things (IoMT) and many other devices. The main causes behind using the HF framework were identified as privacy and security, integrity, traceability, and availability of health records. Additionally, storage issues with transactional data over the blockchain are reduced by the use of the HF framework. This SLR also highlights potential future research trends to ensure the high-level security of health records.

## 1 Introduction

Electronic health records (EHRs) have confidential and sensitive medical information that can be exchanged between healthcare providers, patients, and pharmacists. Clinical information such as past check-up reports, magnetic resonance imaging (MRI) reports, blood reports, and allergies are crucial to patients. This information is available only to patients, physicians, and other authorized users (1). To manage patient information, current EHR techniques are either cloud-based or distributed, which have diverse functionalities and also suffer from financial complications (2). Although EHRs have played a significant role in the healthcare industry, security and privacy concerns have not been comprehensively addressed. Literature reveals that many researchers have used the Ethereum blockchain and cloud-based systems to overcome these concerns (3).

Macdonald et al. (4) presented a comparison of five important blockchain platforms. Ethereum is one of these frameworks. IBM Open Blockchain (OBC), Intel Swatooth Lake, Eris, and BlockStream Side Chain Elements suffer from several limitations. Ethereum has been known as the leader in addressing several challenges, including scalability. Yu et al. (5), in a study, compared the applications of Ethereum and HF frameworks with MultiChain. However, these studies do not consider the applications of blockchain platforms in the healthcare industry (5, 6).

Ethereum and HF are two prominent BCT frameworks. They serve different purposes. The Ethereum platform provides certain features, including public (permission less) and private (permissioned) blockchains. For instance, HF as a decentralized framework is more suitable for permissioned blockchains and can execute distributed applications (Dapps). Both the Ethereum and HF frameworks provide impeccable features to users. However, the HF framework is considered more secure than Ethereum platform (7). Identity, confidentiality, performance, and scalability features of the HF framework are better compared to those of the Ethereum framework (8). Therefore, we choose the HF framework in this study and present its penetration in the healthcare industry.

Recent research explores the importance of hybrid clouds for preserving the security of EHRs (9). A biometric-based schema has been introduced to ensure that legitimate remote users can access the patients’ EHRs (10). Another study shows contrasting results regarding trust, traceability, and security features in the healthcare industry. The BCT offers these services to its remote and distributed users (11). Tampered-proof EHRs are generated by using the BCT. The patient records are verifiable and protected from illegal modifications (12). Ancile, as a blockchain-based approach, uses smart contracts to provide access to obfuscated data and employs cryptography for data security (13).

There have been several reviews undertaken on the topic of BCT and its application in various sectors. The first review article focused on cloud-based, software-defined networks (SDNs) and blockchain-based proposed solutions for the security and privacy of medical information. Proposed approaches to challenges concerning the confidentiality and integrity of massive amounts of medical records were focused on in another review (14). Consequently, a scoping review concluded that provenance, data integrity, and interoperability were the main challenges that could be overcome to improve the performance of BCT in healthcare (15). Similarly, a recent review study highlighted the data security and leakage issues while using BCT on clouds (16). Most recently, a review article analyzed several applications of BCT in the data sharing of EHRs, IoT, and federated learning (17). A systematic literature review (SLR) was undertaken to present an overview of blockchain-based applications. The SLR has a very limited discussion about the HF framework (18). We argue that state-of-the-art approaches regarding the HF framework have received very limited attention, even in some reviews that assess the literature on data security and privacy of health records.

Eventually, the existing literature lacks a comprehensive review of the current blockchain-based approaches using the HF framework to ensure the data security and privacy of health records. Our contribution can be summarized as follows:

1. We present an overview of the state-of-the-art HF framework and its role in securing EHRs in healthcare.
2. We provide a privacy preservation mechanism on blockchain based on the existing literature.
3. We give an overview of the state-of-the-art privacy and security challenges of health data collected from IoMT.
4. We deduce future research directions and opportunities from the HF framework.

The remainder of the article is organized as follows: Section 2 is focused on the materials and methods used for conducting this SLR. Section 3 presents results and discussion on the literature about privacy and security challenges of IoMT data; the HF framework and its application in healthcare; and an analysis of data security and privacy challenges and performance metrics used in the literature. Section 4 provides a discussion on future research directions, while Section 5 gives a conclusion to the present study.

## 2 Materials and methods

A systematic review has been designed using “Preferred Reporting Items for Systematic Review and Meta-Analysis” (PRISMA) guidelines (19).

### 2.1 Search strategy

A search protocol was designed to search articles on topics from popular databases. A search string was proposed by using the appropriate search keywords and terms. A combination of search keywords and terms was used in the ACM digital library, IEEE Xplore, SCOPUS, Springer, PubMed, and Google Scholar databases. The search for literature on the topic was performed between January 2015 and March 2023. The year 2015 was chosen as the HF framework was first introduced as a permissioned blockchain in the year 2015 by the Linux Foundation (Inc., 2023). To promote the research results, search keywords were used that would help in answering the research questions. We used Boolean operators (AND and OR) for search strings as follows:

(Hyperledger Fabric) OR (blockchain framework) AND (health records) OR (electronic health records) OR (patient records) AND (security) OR (privacy)

(Hyperledger Fabric) OR (blockchain framework) AND (internet of medical things) OR (IoMT) AND (security) OR (privacy).

### 2.2 Study selection

**Figure 1:**
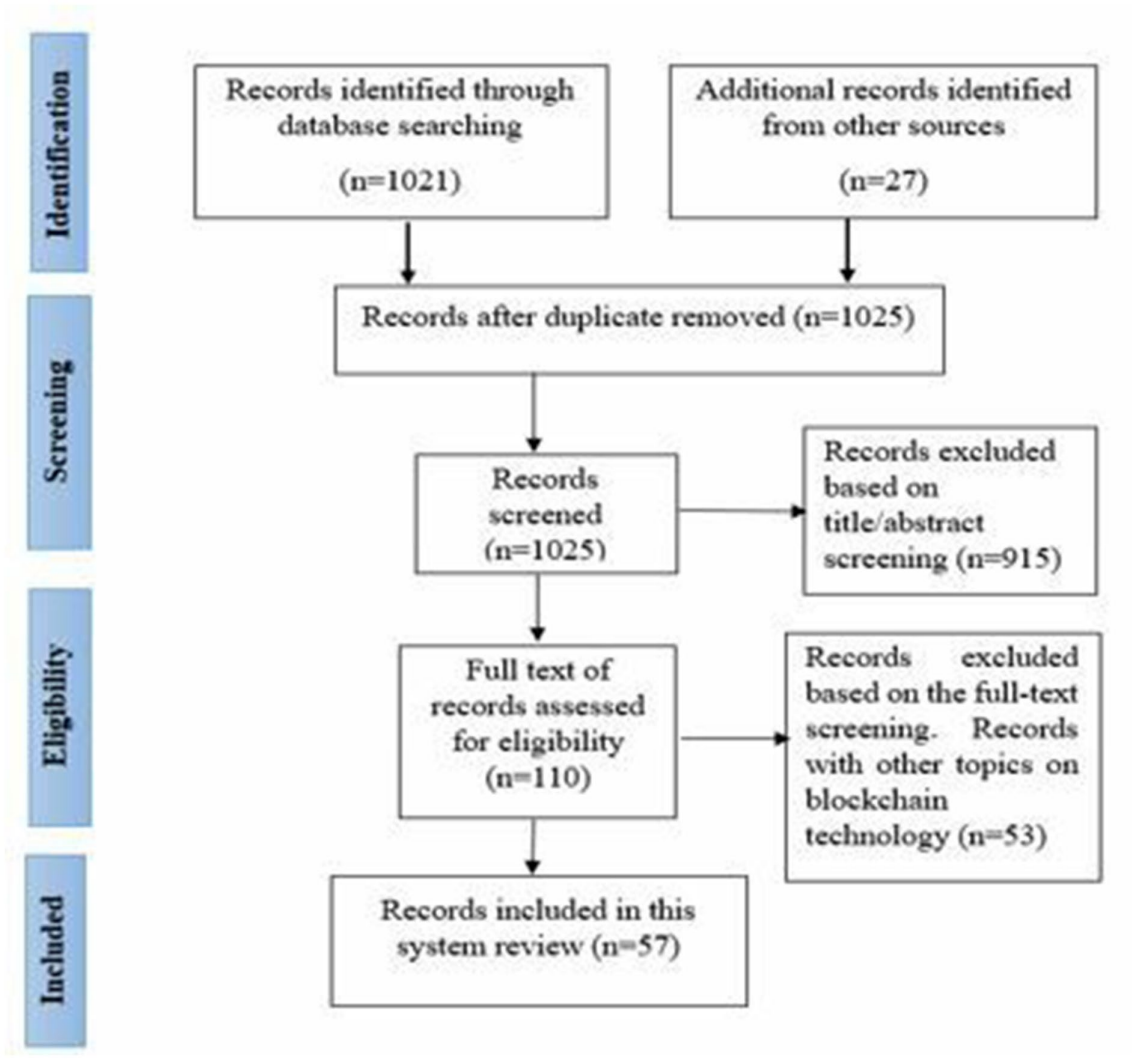
PRISMA flowchart diagram.

The next step of the process was to perform the screening of the relevant studies. The screening process began with the examination of all studies collected from databases in the previous step. We developed a reference list with the help of Endnote X8.0 that was employed to eliminate duplicate studies. The rest of the duplicated documents were removed manually. Titles and abstracts of documents were assessed to determine their relevancy for the current systematic review. Two authors (M.H and I.G) performed the screening of studies using the inclusion and exclusion criteria (Figure 1). Thus, studies that were not clearly focused on the HF framework and its applications in healthcare regarding EHRs were excluded.

### 2.3 Inclusion and exclusion criteria

This systematic review followed the studies’ inclusion and exclusion criteria as follows:

#### 2.3.1 Inclusion criteria

- Studies with sufficient discussion on the HF framework in the healthcare sector
- Studies on HF framework and IoMT
- Studies in English language
- Studies with research findings on the topic

#### 2.3.2 Exclusion criteria

- Studies without the availability of full-length text
- Studies with a focus on other than the HF framework
- Studies with a focus on the HF framework in other than healthcare sectors
- Duplicate studies
- Editorials, short papers, prefaces, readers’ letters, posters, and correspondence

### 2.4 Quality assessment

The quality assessment of the studies was performed according to the guidelines from Kitchenham and Charters (20). This allowed us to assess the relevance of studies to the research topics in this systematic review. Six randomly selected studies were subjected to the process of quality assessment and checked for effectiveness.

Table 1 shows the checklist of quality assessment criteria (QAC) used to qualitatively analyze the collected studies. This checklist was applied to all the studies identified in this systematic review. There were some studies that did not meet one or more of the above-given checklist items, and hence those studies were removed.

**Table 1:** Quality assessment criteria.

Stage#	Topic	Quality assessment criteria
1	HF framework	The study must focus on the use of HF framework or the applications of HF framework to the specific privacy and security problems of health records
2	Context	To elaborate and interpret results accurately, and enough context must be provided by each study.
3	Method	A study must have important details about the use of HF framework to address a specific problem regarding health records
4	Privacy and Security	A study must have enough information about the privacy and security issues of health records
5	Performance	A study must have performance analysis of the HF framework based proposed solutions, and it should be compared with other frameworks
6	Data acquisition	A study must provide the information about the acquired patients’ data, its measurement and reporting.

### 2.5 Data extraction

All studies that had passed the QAC had their data extracted to examine the completeness and accuracy of the information contained in each study. Data from studies were extracted, categorized, and stored in the Excel sheets. The categories of extracted data were as follows:

### 2.6 Contextual information

Information about the purpose of study, problem addressed, and proposed solution is vital for a comprehensive understanding of the research

- **Pros and cons:** The information about the advantages and disadvantages of proposed solutions.
- **Qualitative data:** Final findings and conclusions from the included studies.
- **Quantitative data:** Data observed from experiments and their results. Data also include performance metrics.

## 3 Results and discussion

This section presents the results and their discussion.

### 3.1 Selection results

A total of 1,048 records were identified by using the initial keyword searches on the databases. Of them, 23 duplicate records were removed because they were either conference papers that did not comprehensively explain the research themes, results, and interpretations or short papers that had insufficient information about the topic. Next, 1,021 records were screened based on the title/abstract, following the studies’ inclusion and exclusion criteria. After assessing the title/abstract of the remaining documents, they were reduced to 110 papers that were found eligible for the full-length text analysis. After reading the full-length text documents, 57 papers remained, providing the final set of articles included in this SLR.

**Figure 2:**
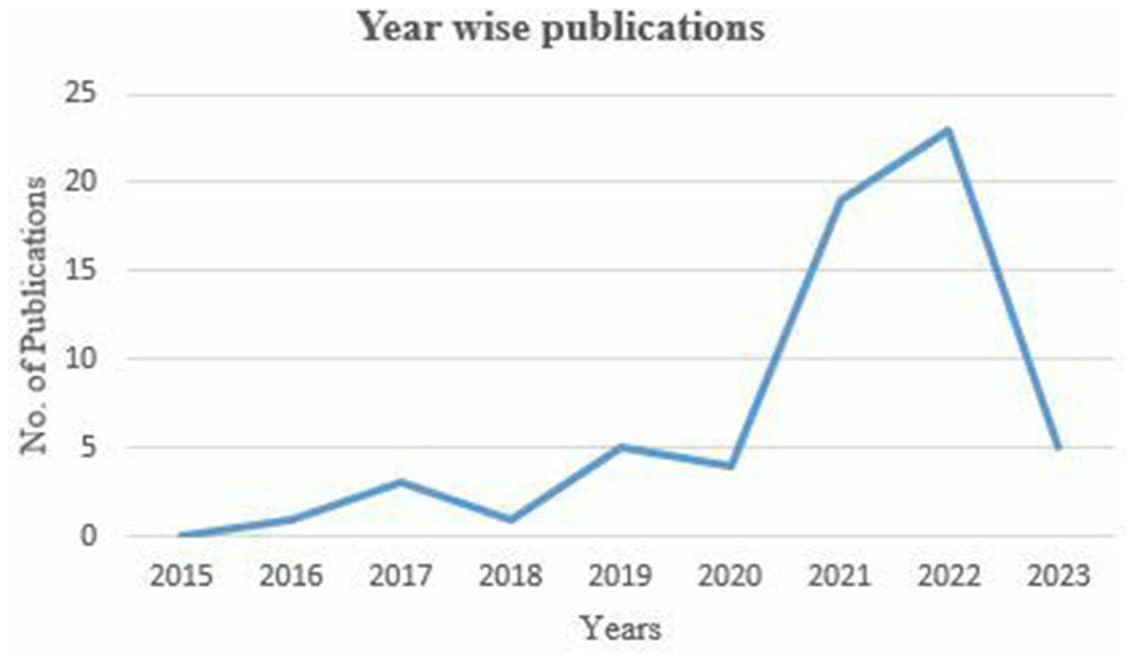
Publications over years (January 2015–March 2023).

Figure 2 presents the year-wise distribution of publications for the chosen period. It can be noticed that, as a result of the search process, including the paper’s inclusion and exclusion criteria, no publications were found in 2015. From 2016 to 2020, this area of research did not receive much attention from scholars. However, Figure 2 shows an interesting point about the rapid increase in publications from January 2021 to February 2023. The increasing trend in published research indicates a significant surge during these years, suggesting that this area of research may become highly sought-after due to its widespread applications across various domains.

### 3.2 Privacy preservation mechanism on Blockchain

The privacy preservation mechanism is based on four aspects. The first aspect is the inclusion of symmetric cryptography and the separation of transaction information from on-chain records. The second aspect involved the digital certificate that ensures the legitimization of organizations on the blockchain. The third aspect is the design of separate multichannels for information distribution. The final aspect isolates the information privacy between the various organizations on the same channel. The HF framework performs two primary functions for data processing. Information processing is based on two criteria: the first criteria involve keeping confidential information and ledgers on a channel for people outside the channel; the second criteria set a scenario where information and ledgers are shared among organizations. Some of them will be able to see the transaction, while others can know about the occurrence of transactions and verify the authentication of transactions (28). However, the information security mechanism becomes weak when several medical institutes lack coordination in healthcare and consensus to determine how data should be utilized or shared when needed (29, 30). Hence, fragmentation problems exist that can be tackled by proposing and implementing a consensus mechanism. To some extent, the implementation of the HF framework has addressed the issue of low transaction efficiency when compared to conventional blockchain architectures such as Bitcoin and Ethereum, which rely on high-power or very complex algorithms to reach consensus (31). Furthermore, the sharing of prevalent health data becomes more challenging when it is shared among stakeholders in its various formats and standards (32). Thus, it becomes hard to examine, aggregate, and share health records in emergency situations.

### 3.3 Data privacy challenges of data collected from the internet of medical things (IoMT)

IoMT is the core application of IoT, which includes remote monitoring and diagnosing health records. Blockchain-enabled IoMT has been deployed to store data at hospital buildings for the provision of real-time monitoring of temperature, air quality, and environmental hygiene (33). Both passive (e.g., tags sensors) and active devices (robots) that can clean hospitals and disinfect can be used. Data collected from these devices can also be used for prescriptive and predictive analysis.

In the context of healthcare, several sensors, such as wearable and off-body sensors, perform sensing of the patient’s body and periodically send health records to a personal digital assistant (PDA). This data passes through the cloud and medical servers, where it is assessed and a prescription is suggested using the patients’ health records (21). Below, Table 2 shows the features of the studied literature on IoMT in healthcare.

**Table 2:** Main features of studied literature on IoMT.

Reference	Main contribution	Blockchain framework	Advantages	Limitations
Chenthara et al. (21)	Proposal and implementation of MedHypChain	HF	Authenticity, scalability, and interoperability of medical data	Proposed approach is complex and requires a higher cost for its implementation in real-world scenarios
Kumar and Chand (22)	Integration of blockchain with MoIT	HF, Quorum, and Corda	Privacy, security, and interoperability of medical data	Contact tracing, location sharing, and supply of medicine during the COVID-19 pandemic are the central issue
Li et al. (23)	Secure sharing of patient records	Not defined	Privacy and security breaches in Internet of Things (IoT). Safe transactions due to asymmetric cryptography technique.	The proposed approach is not efficient in measuring the exact location of patients.
Tiwari et al. (24)	Secure issue of IoT-based health monitoring system	HF	Data integrity, availability, security, and storage issues of transactions	The proposed approach is not evaluated based on performance metrics
Oikonomou et al. (25)	Incorporates machine learning-based anomaly detection in a health monitoring system	HF	A trusted ML-based anomaly detection works better compared to the existing systems	The proposed system is not evaluated based on communication, computational, and storage costs.
Pelekoudas-Oikonomou et al. (26)	Examines the edge learning and IMoT devices	HF and Ethereum	Several features such as fever detection, face mask detection, and in-home cough sound analysis of patients were analyzed	Accuracy of the proposed solution in real subjects is not performed
Rahman Hossain (27)	Efficient and secure transaction of health records	HF	Scalability, traceability, availability, integrity, and confidentiality of patients’ records	Interoperability of the proposed system has not been tested with other IoT frameworks

We identified a few benchmark studies that employed IoMT with blockchain frameworks to make health records more secure and confidential. Innovative research on the integration of IoMT with the BCT is in its infancy stage. As listed in Table 2, studies contributed toward the proposal of approaches that employed only HF (21, 24, 25, 27), or in a combination with other frameworks (22–26). Most recent literature shows that the HF framework is mostly used in this emerging area of research. To assess the quality features of medical records, this SLR identified several quality features and relevant issues studied in the literature.

**Figure 3:**
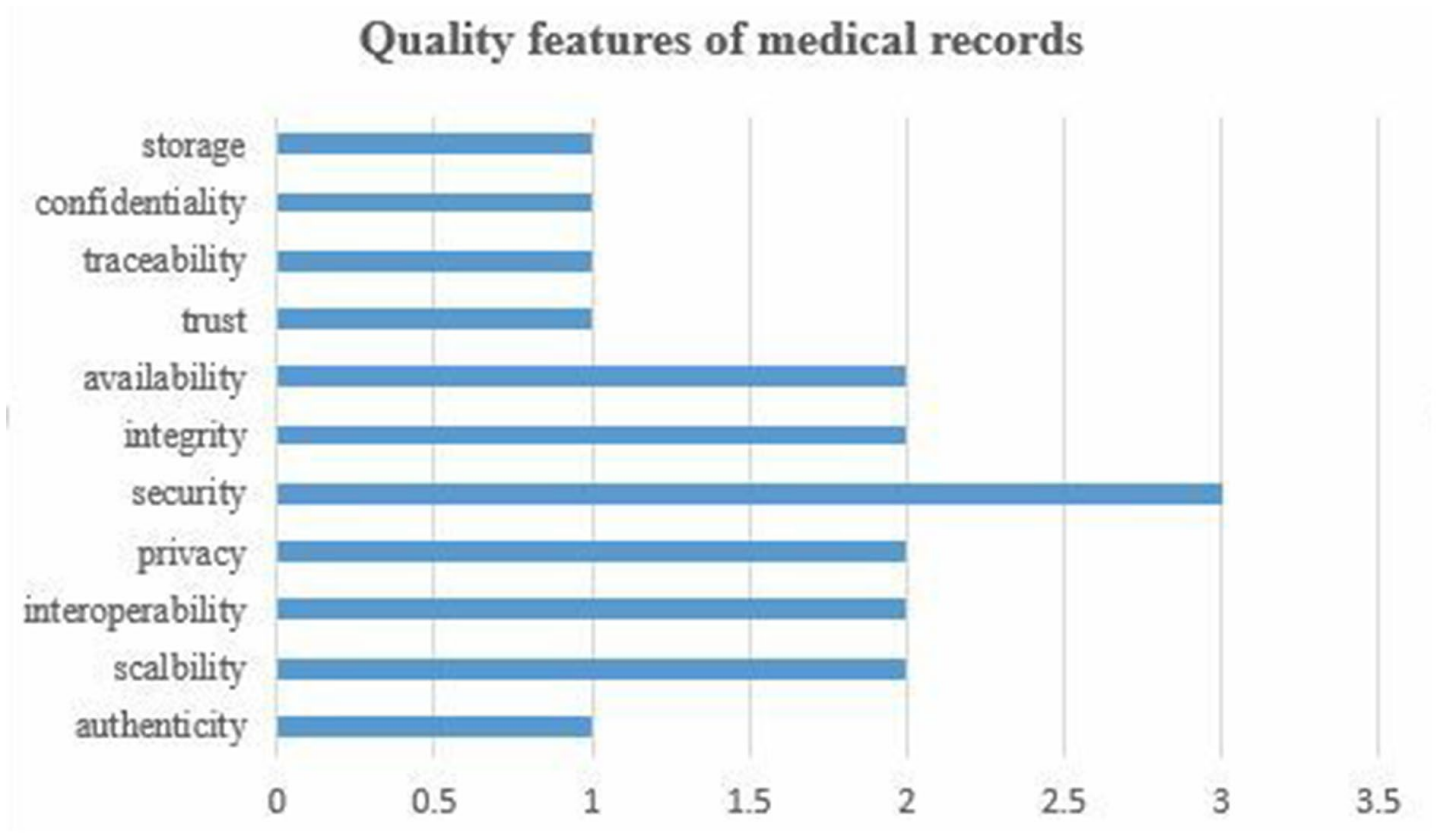
Quality features of medical records.

Figure 3 shows the results on the quality features of medical records. This SLR identified 11 quality features and relevant concerns about them. Security as a quality feature has been widely studied in the literature, followed by availability, integrity, privacy, interoperability, and scalability. The rest of the quality features and their issues have been poorly addressed in the literature. Trend analysis, as shown in Figure 3, indicates that security remained among the priority concerns of scholars. Although several issues regarding quality features were mainly focused on the literature, but proposed approaches suffer from certain challenges in their implementation in real-word scenarios. Implementation cost (21), effectiveness (23), performance evaluation (24, 26), and storage cost (25) are open issues in the emerging area of IoMT integrated with the blockchain frameworks.

### 3.4 Hyperledger fabric platform applications in healthcare

EHRs contain patients’ sensitive information, such as name, address, medical history, social security, and insurance numbers. Patients’ data have worth for stakeholders, and data exposure to the public has adverse consequences. To reduce the impact of patients’ data exposure, the proposed approach used the HF framework to create and test various scenarios for data security (34). The HF framework complies with the General Data Protection Regulation (GDPR) and covers several areas. It has an edge over the other frameworks in preventing cyber-attacks and is best suited for healthcare applications compared to other frameworks. In the following Table 3, we present a summary of the proposed approaches that utilized the HF framework in the healthcare sector.

**Table 3:** Summary of the studied literature on the HF framework and their applications in the healthcare sector.

Reference	Study context	Advantages	Disadvantages
Antwi et al.(35)	Secure exchange of health records	Ensures integrity of health records	Not defined
Margheri et al.(36)	Clinical context of patients’ data is limited	Reading and writing of patient records is in the order of milliseconds	Although throughput is increased without focusing on security
Bhavin et al.(37)	Security and privacy of stakeholders	Proposed approach prevents EHRs from quantum attacks	Proposed approach is not evaluated and compared with other frameworks
Kaur (38)	Security and storage health records	Efficient in securing and storing of health records	No comparison with the other types of blockchain framework
Hang et al. (39)	Data sharing, security and privacy challenges in clinical trial studies	Decentralized approach can cover data sharing, security, and privacy aspects	Several technical open challenges of BCT are not addressed

Most recent approaches using the HF framework have been proposed to secure the exchange of health records and ensure the confidentiality and integrity of records (40–42). The HF framework is focused on monitoring and tracking EHRs on a cloud server, as revealed in a study (43). Health records stored in HF are acquired by the federated server, where they are analyzed and pre-processed. The analyzed and pre-processed data are fed into a module for tailored recommendations, aiding physicians, nurses, and patients in countering health issues. Thus, the survival rate of patients can be increased using the proposed approach. Since the proposed approach integrates blockchain and federated learning, data from emerging COVID-19-like infections require an update to the proposed framework. Before this study, a tamper-resistant mobile health system was developed and evaluated using the HF framework that showed auditability and trusted computing (44). A mobile application using insomnia therapy data was referred to by HF as a blockchain network. This novel implementation led to improved accessibility and data transparency.

### 3.5 Data security and privacy challenges

This section presents an overview of the existing literature on the BCT-based HF framework and applications to resolve data privacy and security problems. Audio data stored in the cloud can be exposed if a decryption method is not implemented. When audio conversations between physicians and patients are stored on clouds, security concerns remain and data leakage can occur. To overcome this issue, a scheme using the homomorphic encryption library (HElib) was proposed and implemented on the “Contabo” cloud platform. The proposed scheme has the potential to increase the computation speed and performance if other open-source libraries such as PALASIDE, SEAL, and HEAAN are used in future studies (45). When the trust level of a cloud storing sensitive data decreases due to a single node failure, the cloud data provider becomes vulnerable to attacks and data theft (46). Table 4 below shows the summary of the studies relevant to the research topic.

**Table 4:** Key problems and their solution via the HF framework.

Study ID/Reference	Problem	Framework	Advantages	Disadvantages
Kumar and Chand (22)	Privacy preservation of patients’ records	HF	Performance analysis using latency, throughput, and execution time is better than the existing approaches	Research shows limitations in showing the implementation of the framework other than HF
Babu et al.(40)	Health records’ security	HF	Enhanced the security, access, scalability, and flexibility of healthcare applications	Blockchain-based security solution suffer higher overheads and regulation compliance
Bai et al. (47)	Sensitive data protection	HF and Ethereum	Higher throughput (≥1,000 TPS) using HF framework	Shows limitation in backing nodes to legitimate all transactions
Stamatellis et al. (48)	Identity identification	HF	Achieves higher throughput than 400 TPS	Only implemented chain-code on the fabric and no wider applications
Sammeta and Parthiban (49)	Exposure to medical records	HF	Provides anonymity and unlink-ability	Idemix technology has technical limitations
Zhao (50)	EMRs security	HF	Better approach than the existing approaches	Learning rate of the proposed approach is the main limitation
Pineda Rincón and Moreno-Sandoval (51)	Scheduling plan for emergency in the medical industry	HF	Optimized solution to prevent attacks while processing transactions	Punishment mechanism for organizations that submit information is not addressed
Hashim et al. (52)	EMRs’ security	HF	Achieved better throughput and latency rates	No additional architectural designs were proposed to make architectural decisions
Shuaib et al. (53)	Interoperability issue	HF	Minimized the average latency between blockchain transfer	Blockchain still suffers scalability and security and needs to be optimized
Azbeg et al. (54)	Denial of service and security attacks on centralized health applications	HF	Better data integrity and security	Trade-off between latency and throughput is not addressed
Pericàs-Gornals (55)	Healthcare systems’ susceptibility to security attacks	Ethereum	Better use of Remix IDE for the protection of patients’ data	No comparison between Hyperledger Fabric and Ethereum framework has been provided in this study
Roehrs et al. (56)	High privacy requirements during COVID-19 pandemic	Ethereum	Control and validation of digital COVID-19 certificates	Filtering the requests for data sharing is not focused in the proposed research.
Zhong et al. (57)	Multiple health records are scattered without integration	HF and Ethereum	99% availability of blockchain-based healthcare applications	Limited in evaluating the content of health records. No use of images as image replicate and more space is used for their storage.
Ravi et al. (58)	Prevention of infected cases during COVID-19 pandemic	HF	Ensures the management of rapidly increasing infected cases	Hospital management is in the preliminary phase and rapid development may suffer quality of service issues

We performed a comprehensive analysis of the pros and cons of the studied literature on the topic. The problems outlined in Table 4 are based on the choices of authors to use blockchain frameworks. Notably, the HF framework was frequently used in research. Out of 14 studies examined, 10 (or 72%) articles employed the HF framework, while 2 (or 14%) articles used the Ethereum framework, and the remaining 2 (or 14%) articles used a combination of two frameworks. Several advantages are brought by the HF framework compared to the Ethereum framework.

**Figure 4:**
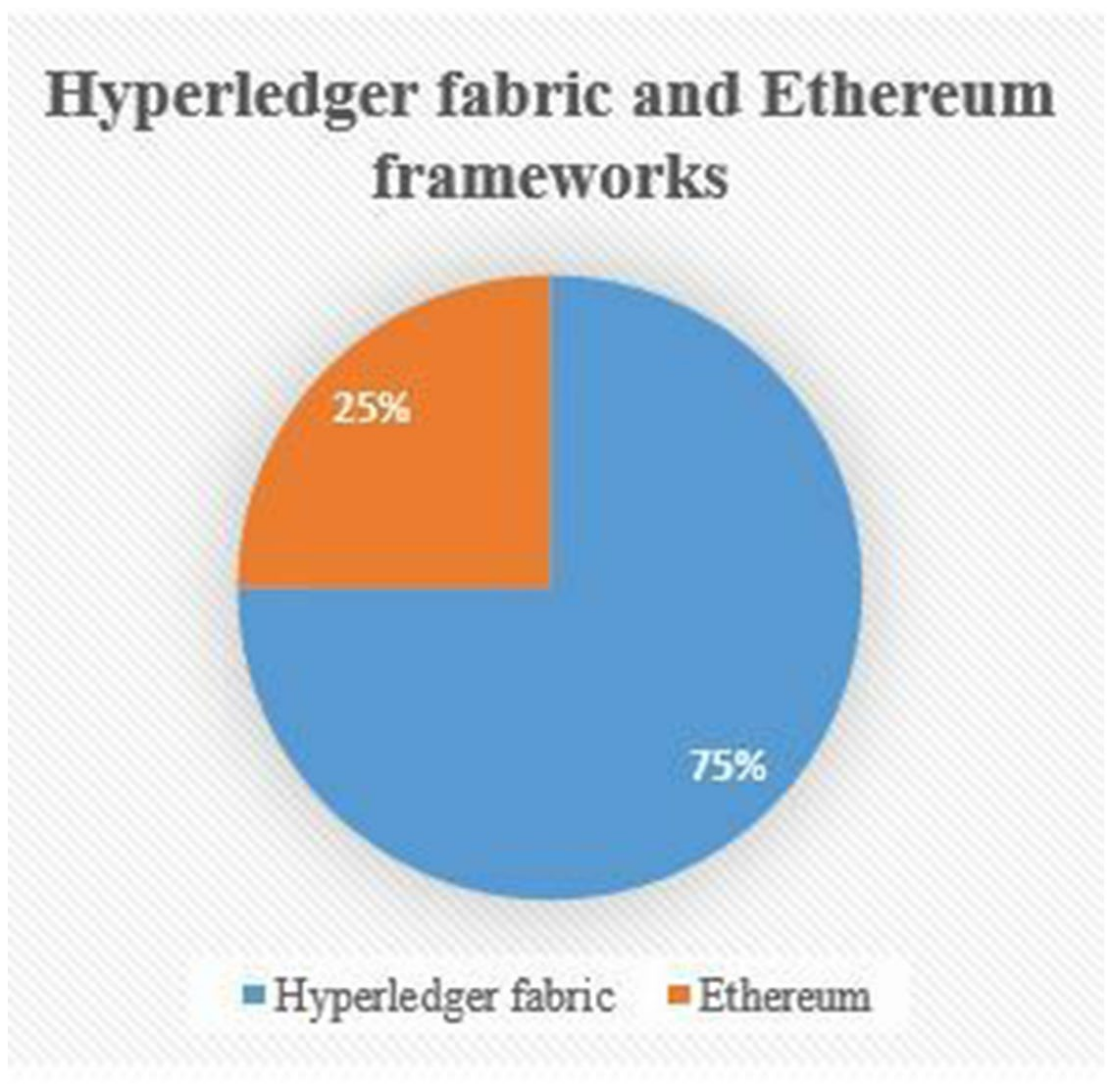
Comparison of the HF and Ethereum framework.

The majority of studies reported the performance aspects of their proposed frameworks. Metrics such as latency, throughput, and execution times were commonly used to highlight the better performance of the HF framework. As listed in the table above, there are core issues related to data privacy and security of health records, effectively prevent potential attacks. Critical systems in healthcare can be protected from external as well as internal attacks by using the applications of the HF framework in healthcare. Figure 4 below gives compelling evidence of the increasing role of the HF framework in tackling the privacy and security challenges of EMRs in the healthcare sector. Comparatively, the Ethereum framework indicates that only a few studies involved Ethereum either alone or in combination with the HF framework to address data security and privacy issues.

**Figure 5:**
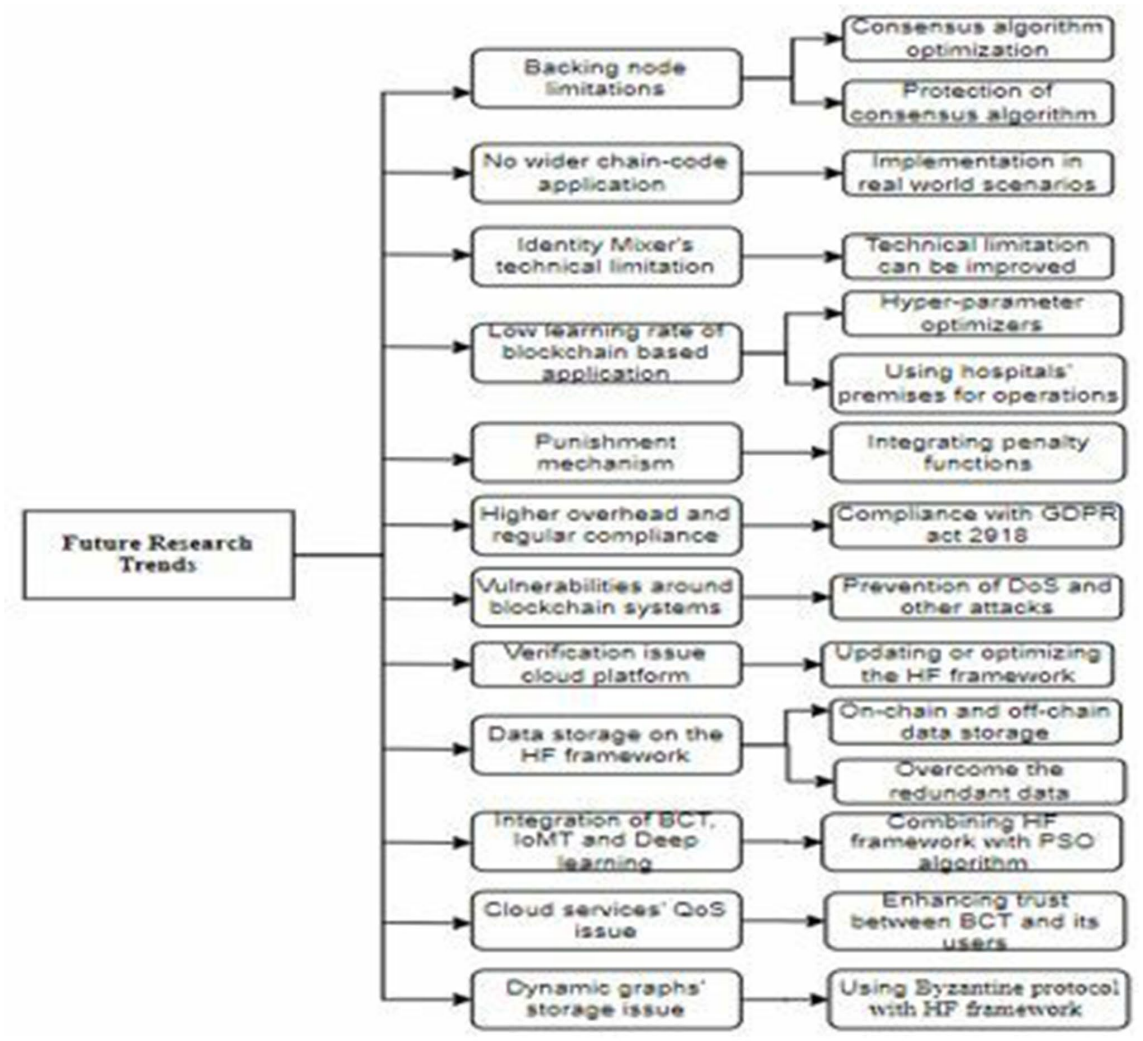
Future research trends.

Handling EMR operations at hospitals using a decentralized platform is one of the primary requirements. This ensures that patients can receive safe and meaningful assistance from healthcare providers and services. The HF framework ensures low resource utilization and high transaction throughput (59). However, computer specifications and blockchain network size are main concerns that may be addressed by expanding the size of networks and deploying the proposed solutions on cloud architecture. However, migrating patients’ data from hospitals to the cloud have its own threat vectors. Sometimes, devices securing medical records become targets of distributed denial-of-service (DDoS) and ransomware attacks (60). To overcome this issue, an interplanetary file system (IPFS) and blockchain can secure and improve data storage (61). Before the emergence of such modern technologies, medical records were either paper-based or stored in conventional databases, which faced issues of security and data duplication. Duplicity of medical records can be prevented by deploying IPFS on networks (Figure 5).

### 3.6 Performance metrics

The key aspect of the blockchain framework is the performance of the proposed solution based on the BCT. Various scholars employed the Hyperledger Caliper as a benchmark method while working on the implementation of BCT. Hyperledger Caliper can be used to generate reports on different performance metrics, including latency, transaction per second (TPS), and execution time (62). The aim of using these performance metrics was to highlight the importance of the HF framework for health records.

Table 5 demonstrates the key metrics and their values to analyze the proposed approaches. It has been pointed out in several studies that the latency of the proposed systems using the HF framework is always lower than the Proof of Work (PoW) protocol, while they exhibit higher throughput values (54). The HF framework-based privacy and security-preserving techniques show a higher level of confidentiality, traceability, and anonymity as compared to the existing approaches. Even these techniques (22) can handle thousands of concurrent transactions in emergency scenarios during the COVID-19 pandemic.

**Table 5:** Key performance metrics analysis.

Study Id/Reference	Name of application	No of users/submitted transactions	Latency (second)	Throughput
Kumar and Chand (22)	Query	10,000	12,067	461 TPS
	Invoke	10,000	12,987	152.1 TPS
Bai et al. (47)	Not defined	1,000	3.6	110 TPS
Stamatellis et al. (48)	Single computer	20,000 success rate	1.04	477 TPS
	Multiple virtual machines	20,000 success rate	1.25	345 TPS
Hashim et al. (52)	Not defined	1,000,000	100	Not defined
Azbeg et al. (54)	Add EHR	10,000	350	7 TPS
	Query EHR	10,000	124	19 TPS
Roehrs et al. (57)	Not defined	10,000	0.449	60 MB/s
Xia et al. (62)	Query batch	250	5.28	30.7
	Create batch (25 TPS)	150	16.30	4.4
	Query batch (25TPS)	250	5.6	34.1
Huang et al. (63)	Not defined	100	1286.53	Not defined
Khan et al. (64)	Select query (Ethereum)	500 records/tx (50,000 records)	35	100 TPS
	Ethereum	500	14 ms	800 Kbps
Xu (65)	Medical services	Not defined	87–95 ms	185 bytes

## 4 Future research trends

BCT, particularly the HF framework, has brought about several advantages. However, this technology does have a number of limitations regarding its applications for securing EHRs in the healthcare industry. These limitations can be deduced as future research directions and opportunities in the realm of blockchain frameworks and their applications in the healthcare sector. Before discussing the identified future research trends, we present a visual representation of the challenges in the figure below.

Ledger Fabric-based techniques show limitations in validating all transactions with backing nodes (47). The consensus algorithm of the HF framework can be optimized to carry a large amount of transactional data. Additionally, the protection of a backing node is another future direction that may be focused on enhancing the transaction speed of the blockchain.

- Ledger fabric technology has only implemented chain code on the fabric, and no broader applications have been proposed in the literature (48). Any user can verify the patients’ records by triggering the chain code. A new user must seek permission to join the chain code. As the chain-code implementation is in its infancy phase, it requires real-world scenarios for testing and implementation.
- Identity Mixer (Idemix) technology, also called zero-knowledge proof (ZKP), provides privacy-preserving features including unsinkability and anonymity (39). However, it has technical limitations, which could be addressed in future releases of the framework.
- The learning rate of the proposed HF framework-based approach is the main limitation (50). This could be overcome by using hyper-parameter optimizers in future studies. Hyper-parameter optimizers are mostly used to solve detection issues using deep learning models (66). By combining the deep learning models with the blockchain, an immutable, secure, and decentralized environment can be enabled for sensitive data. To facilitate the training of deep learning models, hospital premises are a convenient and safe alternative to sharing with the cloud entity (67). This approach helps keep the data under the control of owners and hospital staff, thereby securing health records. Hospitals can be like smart building, and building information modeling (BIM), IoT, and BCTs can be combined to build hospitals such as smart buildings where health data can be stored and managed efficiently and securely (68). This idea could also be applied to other public and private buildings where the safety of humans and other assets, including data, is very significant.
- The punishment mechanism for organizations that submit information is not addressed (51). Therefore, a penalty function can be integrated into future versions of blockchain frameworks to align the proposed techniques with the original requirements.
- Blockchain-based security solutions often face higher overheads and regulatory compliance. A legislative organization may take several years to adopt the technology. The Data Protection Act of 1997 remained the same for this technology proposed in 2018. Cloud computing technology has easier compliance with the GDPR Act 2018, while the implementation of blockchain frameworks struggles with GDPR compliance due to the many resources required to scale up the system.
- Vulnerabilities around the blockchain system can become a major drawback of the system (69). For instance, a blockchain-based system can be attacked during implementation. Poorly written and outdated code leaves vulnerabilities open to exploitation in a decentralized autonomous organization. Vulnerable security attacks on the Hyperledger network can reduce the throughput of the system (70). Additionally, the latency rate increases when these denial-of = service (DoS) attacks are launched.
- A medical cloud platform has been proposed with its several applications in areas of medical institutions, nursing homes, and health monitoring. Compared to conventional big data analysis, it provides users’ data privacy. Moreover, it can facilitate the exchange of encrypted messages related to health records, providing safe, low-cost, and high-quality solutions for smart medical records (71). However, problems with the proposed medical cloud platform can be tackled in future studies. These problems include the efficiency verification of the proposed framework for large-scale applications in cases of real-time requirements. In future studies, the framework’s design can be updated or optimized to improve its universality.
- Data storage is a big challenge for HF and similar blockchain frameworks (72). This challenge is more emphasized when massive IoMT data overloads the blockchain system (23). This challenge is more closely related to the scalability feature of a blockchain system (73). It can be overcome by using on-chain and off-chain data storage. An oracle approach must be undertaken to address the validation of data transferred onto the off-chain facility for each medical operation. Furthermore, tackling the redundant information arriving on blockchain storage and synchronizing the information may help reduce the overload on a blockchain system.
- An earlier study on the integration of artificial intelligence techniques with the BCT aimed to secure data coming from IoT devices (74). An emerging area of research involves the combination of IMoT and BTC with deep learning models (75) to optimize the proposed solutions using these technologies. Optimization is a big challenge for the proposed privacy and security-ensuring techniques (76). A blockchain-based deep learning framework has been presented with two levels of security and privacy (77). This framework is more suitable for various sub-domains in the healthcare sector. However, this framework has the potential to be integrated with the particle swarm optimization (PSO) algorithm and federated learning to enhance privacy and security in health records.
- Optimizing the quality of service (QoS) metrics is the greatest ambition of a cloud service provider and service users. Recent studies have been undertaken on the impacts of BCT on cloud services (76). The primary objective of the proposed approach is to enhance the scheduling and security of the methods used in delivering cloud services. This could be achieved when users show their trust in cloud services (78). Due to the transparency, anonymity, and autonomy features of BCT, this area can be extended in future studies. Trust in BCT is another research area (79), which can be undertaken to make the blockchain more trustworthy by activating its decentralization and privacy-preserving capabilities. This could be used to ensure the users’ trust by protecting them from vulnerable attacks.
- Knowledge graphs can be dynamically created but cannot be stored on chains. It prevents the data from being tampered with (80). However, the use of the PoW algorithm on Ethereum makes the system vulnerable when all nodes simultaneously stop working. This problem can be addressed by shifting toward the HF framework and using the Byzantine protocol as a consensus algorithm (80, 81). Moreover, knowledge graph data, a popular data type, may be used to support authenticated queries, which can be easily manipulated on the blockchain.

## 5 Conclusion

This SLR presented an overview of the HF framework and its applications in securing health records. A SLR protocol as a research method was used to conduct the study. The findings of the SLR indicate that the use of the HF framework is still in its infancy and is gaining research attention from scholars in various domains, including IoT, AI, and cloud computing. Our SLR identified several challenges related to the privacy and security of health records from different sensors and devices. One of the significant challenges is the evaluation of the HF framework-based approaches in real-world scenarios. Major uses of the HF framework were identified as improving the latency, throughput, and execution time of proposed approaches while securing health records. Additionally, this SLR also presented several future research trends that could be explored in upcoming studies.

## Author contributions

MH: Conceptualization, Writing – original draft, Software, Investigation. FA: Investigation, Methodology, Formal analysis, Supervision, Writing – review & editing. SA: Investigation, Methodology, Funding acquisition, Project administration, Validation, Writing – original draft. IG: Formal analysis, Supervision, Writing – review & editing, Resources, Visualization. BM: Project administration, Supervision, Writing – original draft.
